# 1669. Impact of Required Durations of Therapy for Antibiotic Orders at the Time of Order Entry

**DOI:** 10.1093/ofid/ofad500.1502

**Published:** 2023-11-27

**Authors:** Megan R Wright, Jessica Gillon, Sophie E Katz, Ritu Banerjee

**Affiliations:** St. Jude Children's Research Hospital, Nashville, Tennessee; Vanderbilt University Medical Center, Nashville, Tennessee; Vanderbilt University Medical Center, Nashville, Tennessee; Vanderbilt University Medical Center, Nashville, Tennessee

## Abstract

**Background:**

At this large academic children’s hospital, providers were required to select a stop date at the time of antibiotic order entry beginning in May 2022. There currently is limited to no published data surrounding the implementation of this practice and its effect on antimicrobial stewardship. This study aims to determine if requiring stop dates on antibiotic orders impacted antibiotic utilization.

**Methods:**

This was a retrospective, pre- post-interventional study of antibiotic usage at a large academic children’s hospital. The pre-invention period was June – November 2020 and 2021. The post-intervention period was June – November 2022. Normalized days of therapy were based on the National Healthcare Safety Network (NHSN) definition. The following indications were selected: empiric for suspected infection, respiratory tract infection (RTI), intraabdominal/gastrointestinal infection, and skin and soft tissue infection. The secondary objective was to subdivide days of therapy, by drug according to the NHSN classifications. Statistical analysis with the Mann-Whitney U test was completed.

**Results:**

The pre-invention group consisted of 3004 patients and the post-intervention group consisted of 1803 patients. There were no major differences between groups, Table 1. The implementation of stop dates decreased total days of therapy per 1000 days present (DOT) from 251 to 235 (p< 0.001) for the entire sample population as well as DOT for empiric indications (153 DOT vs. 124 DOT, p< 0.001), Table 2. For RTI, there was an increase from 42 DOT to 49 DOT (p=0.011). Further analysis revealed an increase in patients with RTI indications and a decrease in duration of therapy from 6 days to 4 days. There was an overall decrease in use of hospital-onset antibiotics (81 DOT vs. 66 DOT, p< 0.001) as well as vancomycin (33 DOT vs. 28 DOT, p < 0.001), Table 3.

**Figure:**
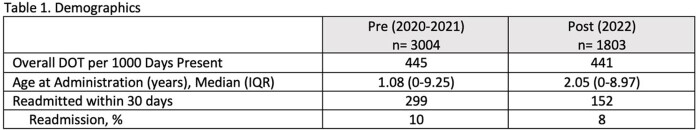

**Figure:**
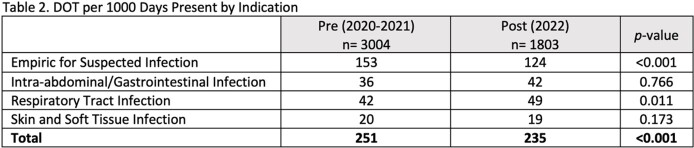

**Figure:**
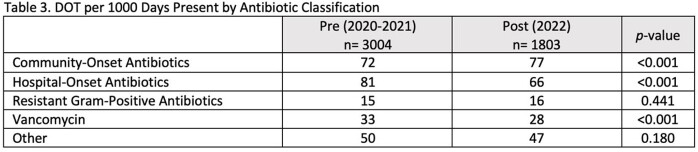

**Conclusion:**

After implementation of stop dates, DOT was significantly lower overall and for empiric indication. Decreased usage in hospital onset antibiotics and vancomycin was also seen. The increase in DOT for RTI can be attributed to the increased prevalence in the post-intervention group. Requiring stop dates at the time of antibiotic order entry was associated with lower antibiotic utilization and is a useful tool for stewardship programs.

**Disclosures:**

**Sophie E. Katz, MD MPH**, Dolly Parton Pediatric Infectious Diseases Research Funds: Grant/Research Support|Optum: Advisor/Consultant|Pfizer: Grant/Research Support **Ritu Banerjee, MD, Ph.D**, bioMerieux: Grant/Research Support|bioMerieux: company is providing partial support for an ongoing trial unrelated to submitted abstract

